# Removal of nucleus pulposus from the intervertebral disc – the use of chymopapain enhances mechanical removal with rongeurs: a laboratory study

**DOI:** 10.1186/1471-2474-8-122

**Published:** 2007-12-13

**Authors:** Lei Dang, Douglas Wardlaw, David WL Hukins

**Affiliations:** 1 School of Engineering, Mechanical Engineering, University of Birmingham, Birmingham, UK; 2 Department of Orthopaedic Surgery, Woodend Hospital, Aberdeen, UK

## Abstract

**Background:**

A laboratory study was conducted, on cadaveric sheep spines to develop an effective procedure for removing as much nucleus as possible from an intervertebral disc with minimal disruption to the annulus. The results of many studies involving removal of nucleus, including chemonucleolysis, using chymopapain, have been published but we are not aware of any previous quantitative studies on procedures for removing as much nucleus as possible from the disc.

**Methods:**

All procedures were performed via a 3 mm trocar. Four procedures were compared: (I) unilateral approach using rongeurs alone, (II) bilateral approach using rongeurs alone, (III) unilateral approach using rongeurs followed by chymopapain and (IV) bilateral approach using rongeurs followed by chymopapain.

**Results:**

The percentages of nucleus removed were: (I) 34%, (II) 41%, (III) 52% and (IV) 75%; there were significant differences between the four sets of results according to ANOVA.

**Conclusion:**

Significantly more nucleus is removed using a bilateral than a unilateral approach; significantly more nucleus is removed if chymopapain is used in addition to rongeurs. A brush is useful in removing strands of nucleus loosened by chymopapain.

## Background

Replacement of the nucleus is an increasingly used procedure for the treatment of chronic low back pain due to symptomatic disc degeneration which is unrelieved by routine conservative care. Extrusion of the implant remains a significant problem and is considered to be related to insufficient removal of the nuclear material [1-3]. Several in vitro studies have been published to develop implants or investigate possible implant materials [4-6]. Chymopapain is an enzyme that has been used successfully in the treatment of herniated nucleus, as alternative [7-9] or as an adjunct [10] to surgery. The published complications following nucleus removal by chymopapin are significantly lower than following surgery [11,12]. This paper describes in vitro methods developed to remove as much nucleus as possible from discs for studies of nucleus replacement. The experiments were carried out on sheep spines since they are increasingly being used as models for human spines in biomechanical studies [13-15].

## Methods

### Study Design

Two spines, each of which consists of seven discs, were used for each of the following procedures: (I) unilateral approach using rongeurs alone, (II) bilateral approach using rongeurs alone, (III) unilateral approach using rongeurs followed by chymopapain and (IV) bilateral approach using rongeurs followed by chymopapain.

### Materials

Sheep lumbar spines were obtained from an abattoir (McIntosh McDonald, Aberdeen). Seven lumbar discs were removed from each spine by sawing through the mid-transverse plane of each vertebra. Pedicles and transverse processes were removed by sawing close to the vertebral body. All extraneous soft tissue (muscle, ligament and fat) was removed to leave an intact intervertebral disc attached to half a vertebra on either side. The resulting segments were wrapped in tissue soaked in physiological saline (9.5 g/L NaCl in de-ionised water) to keep them moist, surrounded by two polythene bags (which were heat-sealed) and stored in a freezer at -40°C. This condition has been shown to have no affect on chemical composition of the disc [15]. Before use, they were thawed overnight at 5°C.

### Rongeurs

Each specimen was secured using a vice. A 3 mm trocar was made for postero-lateral access to the nucleus (with an entrance angle of 40–60° to the antero-posterior axis), following standard procedure for percutaneous nucleotomy [16,17]. The trocar was pushed and rotated simultaneously, to perforate the annulus, until a sudden resistance was felt. A 3 mm straight rongeur (Smith & Nephew Richards Inc, Memphis TN, USA) and a 2 mm upbiting rongeur (Smith & Nephew Richards Inc, Memphis TN, USA) were used to remove the nucleus. The procedure was continued until no more nucleus could be removed. The disc was then bisected (in the transverse plane), photographed, and the remaining nucleus removed with rongeurs. Each sample of nucleus removed (before and after bisection) was placed on a weighed piece clingfilm, wrapped and re-weighed; the former was the mass of nucleus removed by the procedure, the latter was the total mass of nucleus.

### Chymopapain

**Figure 1:**
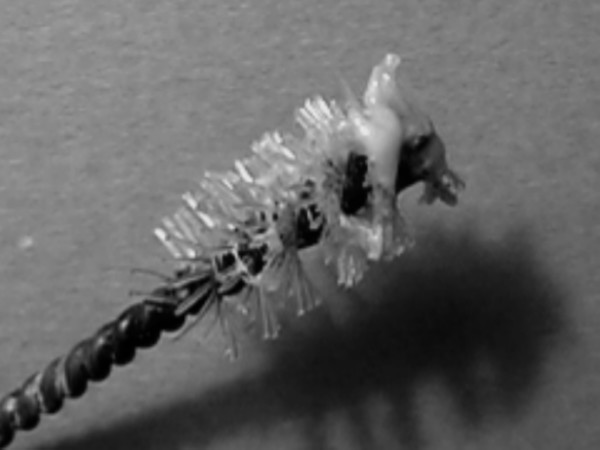
Strands of nucleus adhering to the brush used after injection of chymopapain.

As much nucleus as possible was removed using rongeurs, and the nucleus removed and weighed as described previously. The specimen was double-wrapped in Clingfilm (to prevent drying) and placed in a 37°C oven for 100 minutes, until it was heated to body temperature. Clingfilm had been shown to prevent detectable water loss in preliminary test. The specimen was then weighed and a needle used to introduce fissures in the remaining nucleus, before injection of chymopapain. The reason for introducing fissures was that chymopapain flows down fissures in prolapsed discs [18]. Chymopapain (30 units; Sigma-Aldrich, Poole, Dorset, UK) was dissolved in de-ionised water (0.1 cm^3^) at room temperature and used immediately. The dose of chymopapain was based on the work of Kudo et al. [19] which showed it produced no adverse effects on living dogs. This solution was slowly injected into the nuclear cavity using a syringe. The specimen was then returned to the oven for 10 minutes, to keep the chymopapain solution and tissue at 37°C. A bottle brush with flexible nylon bristles (trimmed to an overall diameter of about 10 mm) was used to remove strands of nucleus which were freed by chymopapain (Figure 1). Excess liquid was removed by suction through a plastic tube (2 mm internal diameter) using a pump (model TS400D, Ohaus Corporation, Florham Park NJ, USA). The surface of the specimen was then dried with tissue paper to remove any remaining solution; if excess fluid had not been removed, its mass would have been attributed to remaining nucleus and so given an under-estimate of the mass of nucleus removed. The specimen was then re-weighed. The reduction in mass of the specimen before and after injection of chymopapain was the mass of the nucleus removed by chymopapain. The specimen was then bisected and photographed as before.

### Calculations

The proportion of nucleus removed was the mass of nucleus removed by the procedure divided by the total mass of nucleus. These proportions were compared by ANOVA using Minitab (release 13, Minitab Inc, State College PA, USA) to determine any statistically significant differences between spines and methods for removing the nucleus.

## Results

The mean percentage of the nucleus removed by each procedure was: (I) 34 ± 2%, (II) 41 ± 2%, (III) 52 ± 3% and (IV) 75 ± 8%. ANOVA showed significant differences between I and II (p < 0.05), II and III (p < 0.05) and III and IV (p < 0.05). However, the spine from which the data were obtained had no significant influence (p < 0.05) on the results.

**Figure 2:**
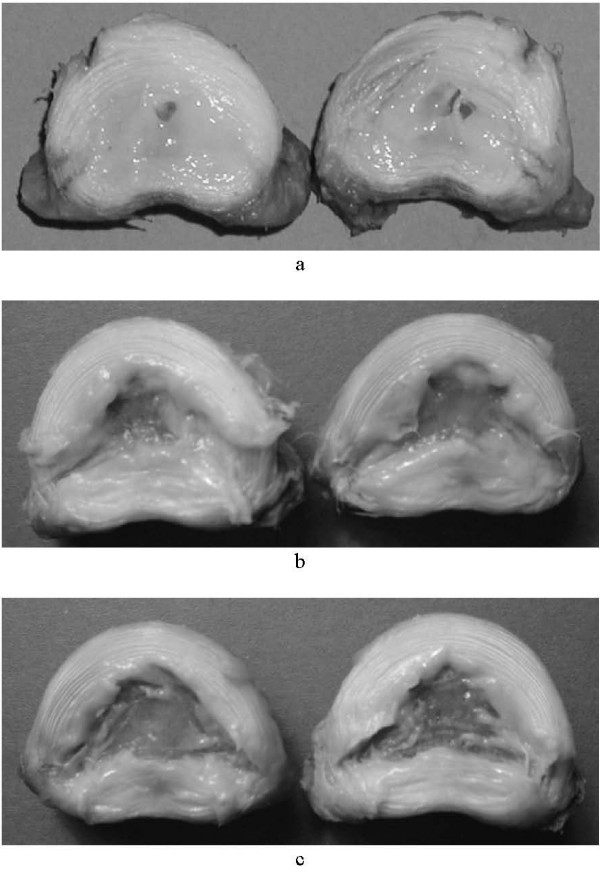
Specimens bisected in the transverse plane after (A) unilateral nucleus removal with ronguers only and (B) bilateral nucleus removal with ronguers and chymopapain; (C) shows the same disc as (B) but the residual nucleus has been removed after bisection.

Figure 2A shows the two halves of a specimen in which the nucleus was removed by the least successful procedure (I) that involved unilateral application of rongeurs alone. Figure 2B shows the two halves of a specimen in which the nucleus was removed by the most successful procedure (IV) that involved bilateral application of rongeurs followed by chymopapain injection. Comparison with Figure 2C, in which the remaining nucleus was removed from the same specimen as in Figure 2B, after bisection, provides a qualitative assessment of how much nucleus it is possible to remove from an intact disc.

## Discussion

These experiments show that a bilateral approach leads to a significantly greater proportion of nucleus being removed than a unilateral approach. This result is not surprising. However, we have shown that a unilateral approach leads to removal of only about one-third of the total mass of the nucleus; Figure 2A shows qualitatively how little material can be removed.

The use of chymopapain, following the use of rongeurs, leads to significantly more nucleus being removed than if rongeurs alone are used. A bilateral approach leads to about three-quarters of the nucleus being removed. This is demonstrated, qualitatively, by Figure 2B. However, comparison of Figures 2b and 2c shows that appreciable nucleus still remains after this procedure. In the course of preliminary experiments, it was found that strands of nucleus isolated by chymopapain, could readily be removed on the bristles of a brush (Figure 1). This finding is consistent with the observation that chymopapain leads to the nucleus being only loosely bound together, and the effects of the action of chymopain continue for up to 48 hours after injection into the disc [18].

It is proposed that some form of brush should be considered as an instrument for removal of nucleus in surgical procedures, following the injection of chymopapain. The design of such a brush should be aided by a preliminary risk analysis [20].

The design of the experiment could be criticised in that the rongeurs were designed for a human disc but were used in a sheep disc, which is much smaller. However, no difficulty was encountered in manipulating the rongeurs, of the size used, within the nucleus of the sheep disc. The main conclusions: that a bilateral approach is more successful than a unilateral approach and that chymopapain can remove additional nucleus, after the use of rongeurs, are then likely to apply to larger human discs. The experiments reported here quantify the percentage of nucleus which it is feasible to remove and the photographs of the bisected discs provide a qualitative impression of the effects of these procedures on the disc nucleus. They show that the use of chymopapain in addition to mechanical removal by means of surgical rongeurs, can increase the amount nucleus removed. Further refinement is required for the implantation of a prosthetic disc nucleus in the human spine.

## Conclusion

This study indicates that significantly more nucleus can be removed from a disc using a bilateral rather than a unilateral approach; a unilateral approach using rongeurs alone leads to removal of only about one-third of the nucleus. Rongeurs, followed by chymopapain, lead to removal of significantly more nucleus than when rongeurs are used alone. The most effective method is a bilateral approach using rongeurs followed by chymopapain where about three-quarters of the nucleus can be removed. This approach can create sufficient space for the implant insertion. A brush, which can be inserted through a trocar, assists in the removal of strands of nucleus that are loosened by chymopapain.

## Completing interests

The author(s) declare that they have no competing interests.

## Authors' contributions

DL carried out the tests and statistical analysis, and participated in drafting the manuscript. DW and DH participated in the design of the study and helped to draft the manuscript. All authors read and approved the final manuscript.

## Pre-publication history

The pre-publication history for this paper can be accessed here:


